# Therapeutically targeting guanylate cyclase‐C: computational modeling of plecanatide, a uroguanylin analog

**DOI:** 10.1002/prp2.295

**Published:** 2017-03-12

**Authors:** Andrea Brancale, Kunwar Shailubhai, Salvatore Ferla, Antonio Ricci, Marcella Bassetto, Gary S Jacob

**Affiliations:** ^1^ School of Pharmacy and Pharmaceutical SciencesCardiff UniversityCardiffUnited Kingdom; ^2^ Synergy PharmaceuticalsNew YorkNew York

**Keywords:** guanylate Cyclase‐C, linaclotide, molecular dynamics, plecanatide, uroguanylin

## Abstract

Plecanatide is a recently developed guanylate cyclase‐C (GC‐C) agonist and the first uroguanylin analog designed to treat chronic idiopathic constipation (CIC) and irritable bowel syndrome with constipation (IBS‐C). GC‐C receptors are found across the length of the intestines and are thought to play a key role in fluid regulation and electrolyte balance. Ligands of the GC‐C receptor include endogenous agonists, uroguanylin and guanylin, as well as diarrheagenic, Escherichia coli heat‐stable enterotoxins (ST). Plecanatide mimics uroguanylin in its 2 disulfide‐bond structure and in its ability to activate GC‐Cs in a pH‐dependent manner, a feature associated with the presence of acid‐sensing residues (Asp2 and Glu3). Linaclotide, a synthetic analog of STh (a 19 amino acid member of ST family), contains the enterotoxin's key structural elements, including the presence of three disulfide bonds. Linaclotide, like STh, activates GC‐Cs in a pH‐independent manner due to the absence of pH‐sensing residues. In this study, molecular dynamics simulations compared the stability of plecanatide and linaclotide to STh. Three‐dimensional structures of plecanatide at various protonation states (pH 2.0, 5.0, and 7.0) were simulated with GROMACS software. Deviations from ideal binding conformations were quantified using root mean square deviation values. Simulations of linaclotide revealed a rigid conformer most similar to STh. Plecanatide simulations retained the flexible, pH‐dependent structure of uroguanylin. The most active conformers of plecanatide were found at pH 5.0, which is the pH found in the proximal small intestine. GC‐C receptor activation in this region would stimulate intraluminal fluid secretion, potentially relieving symptoms associated with CIC and IBS‐C.

**Abbreviations**
CIC: chronic idiopathic constipation
FGID: functional gastrointestinal disorder
GC‐C: guanylate cyclase‐C
GI tract: gastrointestinal tract
IBS‐C: irritable bowel syndrome with constipation
RMSD: root mean square deviation
ST: family of heat stable enterotoxin produced by enterotoxigenic *Escherichia coli* that include STh and STp
STh: 19 amino acid member of ST family

## Introduction

Chronic idiopathic constipation (CIC) and irritable bowel syndrome with constipation (IBS‐C) are two of the most common conditions affecting the gastrointestinal (GI) tract, creating a burden on healthcare resources and leading to significant negative impact on quality of life (Heidelbaugh et al. 2015). These disorders are characterized by diminished stool frequency, straining and abdominal pain (IBS‐C) or discomfort (CIC). CIC alone affects 14% of the North American population, is challenging to treat and poses a significant burden on health resources (Suares and Ford 2011).

Guanylate cyclase‐C (GC‐C) receptors play a crucial role in the maintenance of normal bowel function and thus have potential as a target for pharmaceutical intervention in to treat numerous functional gastrointestinal disorders. The GC‐C receptor is a membrane‐spanning protein uniformly expressed along the epithelial brush border throughout the intestine (Forte 1999). Recently plecanatide and linaclotide have been developed for the treatment of two of these disorders, CIC and IBS‐C (Shailubhai et al. 2013).

The GC‐C receptor is activated by its endogenous peptides uroguanylin and guanylin that differentially bind to the receptor in the varying pH environments found along the GI tract (Fan et al. 1997). Uroguanylin is primarily expressed and preferentially binds GC‐C receptors in the slightly acidic (pH 5‐6) regions of the duodenum and jejunum (Forte 1999). Guanylin is primarily expressed in the ileum and colon, activating GC‐C receptors under more basic conditions (pH 7 ‐8) (Kita et al. 1994). Binding of uroguanylin or guanylin to the GC‐C receptor initiates a signaling cascade leading to accumulation of intracellular cyclic guanosine monophosphate (cGMP) (Vaandrager et al. 1997), which helps maintain fluid and electrolyte balance, promotes visceral analgesia, and reduces inflammation in the GI tract (Hughes et al. 1978; Pitari 2013; Shailubhai et al. 2015; Hanning et al. 2014).

The overlapping yet distinct activities of guanylin and uroguanylin suggest that the tight and tunable regulation of GC‐C receptors is essential for proper GI function, a feature that becomes readily apparent by the consequences of dysfunctional GC‐C activity (Whitaker et al. 1997). Overactivation of GC‐C receptors by the *E. coli* enterotoxin (STh) triggers an uncontrolled release of electrolytes and water into the intestinal lumen resulting in diarrhea (Brierley 2012).

Studies have shown STh to be 10 times more potent than uroguanylin and 100 times more potent than guanylin in binding to GC‐C receptors (Hamra et al. 1993). The X‐ray structure of STh reveals that the molecule is locked into a constitutively active, right‐handed spiral formation stabilized by three intrachain disulfide bridges in a 1‐4/2‐5/3‐6 pattern (Gariepy et al. 1987; Ozaki et al. 1991; Shimonishi et al. 1987). Unlike STh, the endogenous peptides have only two disulfide bridges which likely results in their improved flexibility; Klodt et al. (1997). The flexibility afforded by absence of a third disulfide bridge allows uroguanylin and guanylin to adopt two topological isoforms (A and B) of which only the A form is biologically active (Marx et al. 1998; Skelton et al. 1994). The pH‐dependent activity of uroguanylin is linked to two charged acid‐sensing aspartic acid residues on its N‐terminus (Hamra et al. 1997). The absence of these residues within STh allows it to bypass pH checkpoints governing GC‐C receptor activation, allowing for supraphysiological activation of GC‐C along the length of the small intestine and colon (Hamra et al. 1997).

**Figure 1:**
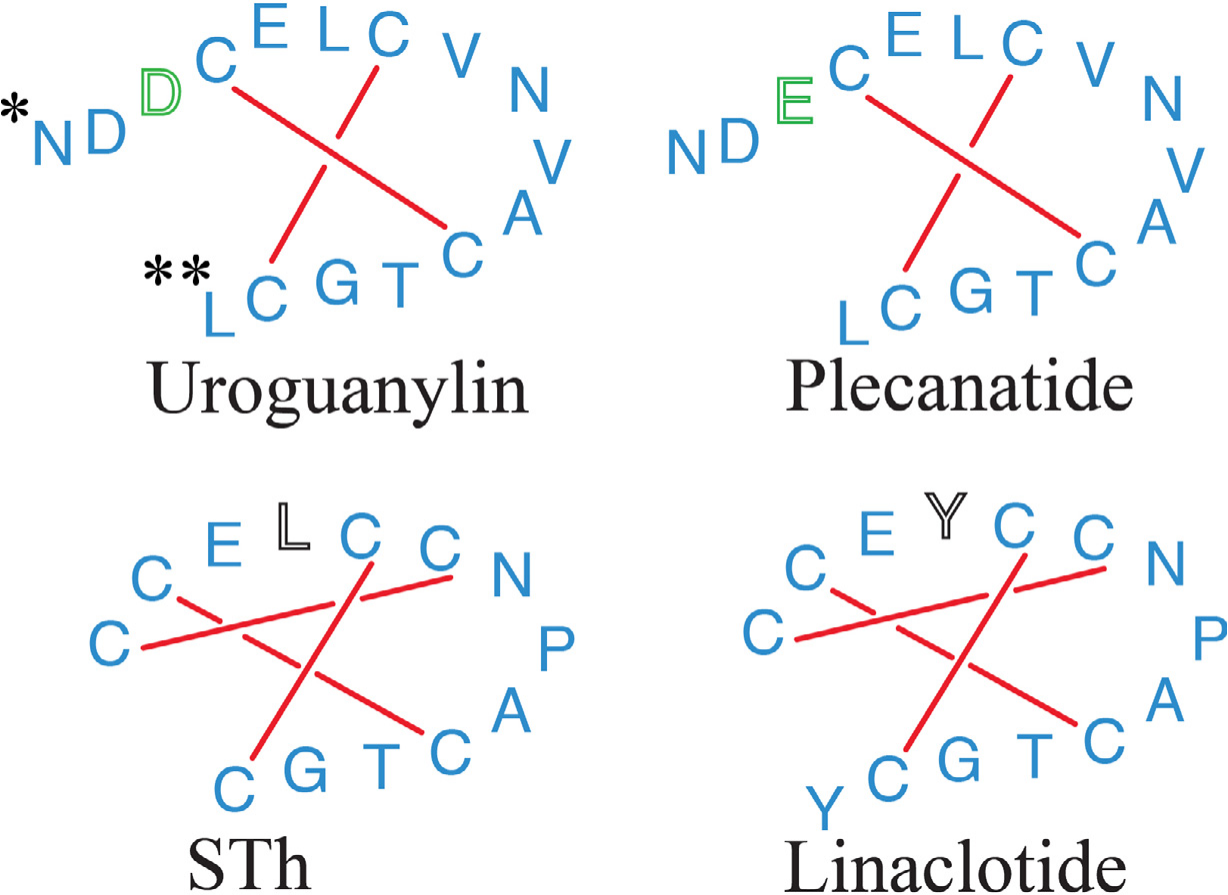
Amino acid structures of guanylate cyclase‐C receptor agonists examined in this study. Synthetic analogs linaclotide and plecanatide share similar amino acid sequences with GC‐C agonists STh and uroguanylin, respectively. Plecanatide, like uroguanylin contains two pH‐sensing residues on its N‐terminus. The pH‐sensing residue (aspartatic acid, D) of uroguanylin is replaced with another pH‐sensing residue (glutamic acid, E) in plecanatide (green). In this study, the pH‐sensing aspartic acid and glutamic acid residues of plecanatide were differentially protonated to reflect pH values 2.0 (Asp2, Glu3), 5.0 (Asp2‐,Glu3; Asp2, Glu3‐) & ≥7.0 (Asp2‐, Glu3‐). Simulations of the crystal structure of STh used a truncated version of the full toxin, comprised of residues 5–17 of the full heat‐stable enterotoxin protein representing the core bioactive pharmacophore of peptide. (A). STh and linaclotide both lack pH‐sensing residues on their N‐terminal ends and are stabilized into a constitutively active conformer by the presence of 3 disulfide bonds. Structurally, the peptides differs by the replacement of leucine (L) in STh with tyrosine (Y*) in linaclotide.

Pharmacologic agonists that mimic the activity of known GC‐C agonists have been developed to treat patients with CIC and IBS‐C. Linaclotide, a synthetic analog of STh, is available for the treatment of CIC and IBS‐C (Lembo et al. 2011). Like STh, linaclotide has three disulfide bonds and demonstrates pH‐independent activation of GC‐C receptors (Fig. 1) (Busby et al. 2010).

Plecanatide is a recently developed GC‐C agonist and uroguanylin analog that is currently in Phase 3 trials for CIC and IBS‐C (Synergy Pharmaceuticals Inc 2015a,b). Plecanatide is an orally administered, pH‐dependent agonist of the GC‐C receptor that shares the structural and physiological characteristics of uroguanylin. Plecanatide has two disulfide bonds, similar to uroguanylin, and contains two acidic N‐terminal amino acids allowing the two molecules to maintain the same pH‐dependent binding characteristics (Fig. 1) (Shailubhai et al. 2013). Given the experimental challenges of studying the unique characteristics of its pH‐sensitive structure, computational methods were employed to characterize behavior of plecanatide at various pH states.

Molecular dynamics (MD) simulations compared the flexibility and conformation of plecanatide and linaclotide. As expected, linaclotide was shown to adopt rigid conformations, which do not deviate significantly from the structure of STh. In contrast, plecanatide showed greater flexibility and an ability to adopt several conformations which vary in response to pH values (2.0, 5.0 and 7.0). Active plecanatide conformations were more similar to uroguanylin than to the STh peptide, suggesting that plecanatide has a similar pH‐dependent activity profile as uroguanylin and that plecanatide's activity can be differentially regulated in the GI tract, with higher activity in the more acidic proximal small intestine and lower activity in the more basic distal small intestine and colon.

## Materials and Methods

### Peptide preparation

The NMR structures of uroguanylin (PDB ID: 1UYA) and the crystal structure of STa (PDB ID: 1ETN) (Fig. S1) were used as starting points for the MD simulations (Ozaki et al. 1991). The STa family of heat stable peptides includes STh and STp (Nataro and Kaper 1998). The series of experiments used in this study used the STh sequence as reference.

Structural models of plecanatide, linaclotide, and STh were built by appropriately modifying the uroguanylin and STa sequences, respectively, using the builder tools in molecular operating environment [(MOE 2015.4) www.chemcomp.com]. (Molecular Operating Environment 2015).

Simulations and root mean standard deviation (RMSD) values of linaclotide and each configuration of plecanatide were compared to the enteric pathogen, STh, which was selected as the reference peptide because of its enhanced affinity for the GC‐C receptor compared to other agonists. The structural rigidity of STh confers one main conformation that would bind the receptor. Similarities to this one conformer would reflect a drugs ability to activate GC‐C.

### MD simulations

All MD simulations of plecanatide, STh, and linaclotide were performed and analyzed using the GROMACS 4.5 simulation package (Hess et al. 2008). MD simulations of the structure of STh used residues 6–18 of the full heat‐stable enterotoxin protein (C‐C‐E‐L‐C‐C‐N‐P‐A‐C‐T‐G‐C), considered to be the pharmacophore of the molecule required for maximum biological activity (Ozaki et al. 1991; Yoshimura et al. 1985).

Four protonation states of plecanatide were modeled by placing the appropriate charge on Asp2 and Glu3, reflecting the most abundant species at three pH environments: pH 2.0 (Asp2, Glu3), pH 5.0 (Asp2‐, Glu3; Asp2, Glu3‐), and pH 7.0 (Asp2‐, Glu3‐) (Fig. 1 circle). The initial structure of each peptide was placed in a cubic box with TIP 3P water and energy minimized using a Steepest Descent Minimization Algorithm. The system was equilibrated via a 50 ps MD simulation at 310 K in a NVT canonical environment followed by an additional 50 ps simulation at constant pressure of 1 atm (NPT). After the equilibration phases, a 500 ns MD simulations were performed at constant temperature (310 K) and pressure with a time step of 2 fs. The system energy and peptide spatial coordinates (trajectory file) were stored every 300 ps for further studies. All MD simulations were run in triplicate for each peptide.

After removing the first 100 ns, considered as system stabilization time, the remaining 400 ns of the MD trajectory of every single run for each group of triplicate experiments were combined using the tricat function. The combined trajectories (3999 frames) were examined using the g‐cluster function, setting gromos as the clustering method with an RMSD cut‐off of 0.1 nm. The different structural cluster groups were obtained as a pdb file. Cluster groups representing at least 10% of the total population for each peptide were selected as the most representative structure for that peptide.

### RMSD comparisons

The representative cluster conformations of the different peptides were used for the RMSD comparison against the main conformation of the STh cluster.

RMSD comparisons were performed using MOE 2015.4 with the major STh cluster structure serving as a reference for the superimposition of other peptide conformations.

## Results

### MD clustering

Table 1 shows the results obtained from the structural cluster calculations. From this data it is possible to appreciate how flexible plecanatide is, compared to STh and Linaclotide. The latter peptides generated more populated cluster than any of the plecanatide forms. RMSD analysis of each MD simulations calculated against the most representative clusters also confirm this observation (Fig. S1).

**Table 1:** Representative cluster analysis

Compound and test condition	Cluster size^a^	% On the total frames^b^	Average RMSD (Å) ± SD^c^
STh	2273	56	0.1252 ± 0.0821
Linaclotide	3592	89	0.0715 ± 0.0421
Plecanatide – pH>7.0	1108	28	0.1827 ± 0.0862
Plecanatide – pH 5.0 (Asp‐/Glu)^d^	524 497 446	13 12 11	0.3208 ± 0.1325 0.2425 ± 0.1044 0.1252 ± 0.1107
Plecanatide – pH 5.0 (Asp/Glu‐)	610	15	0.2885 ± 0.1363
Plecanatide – pH<2.0	709	18	0.2115 ± 0.0825

a The cluster size refers to the number of frames that are forming the cluster. The total number of frames is 3999.

b The % is calculated on the total number of frames.

c The average RMSD is calculated against the most representative cluster conformation for each peptide.

d Three representative clusters were obtained for this peptide.

### MD simulations of STh and linaclotide

**Figure 2:**
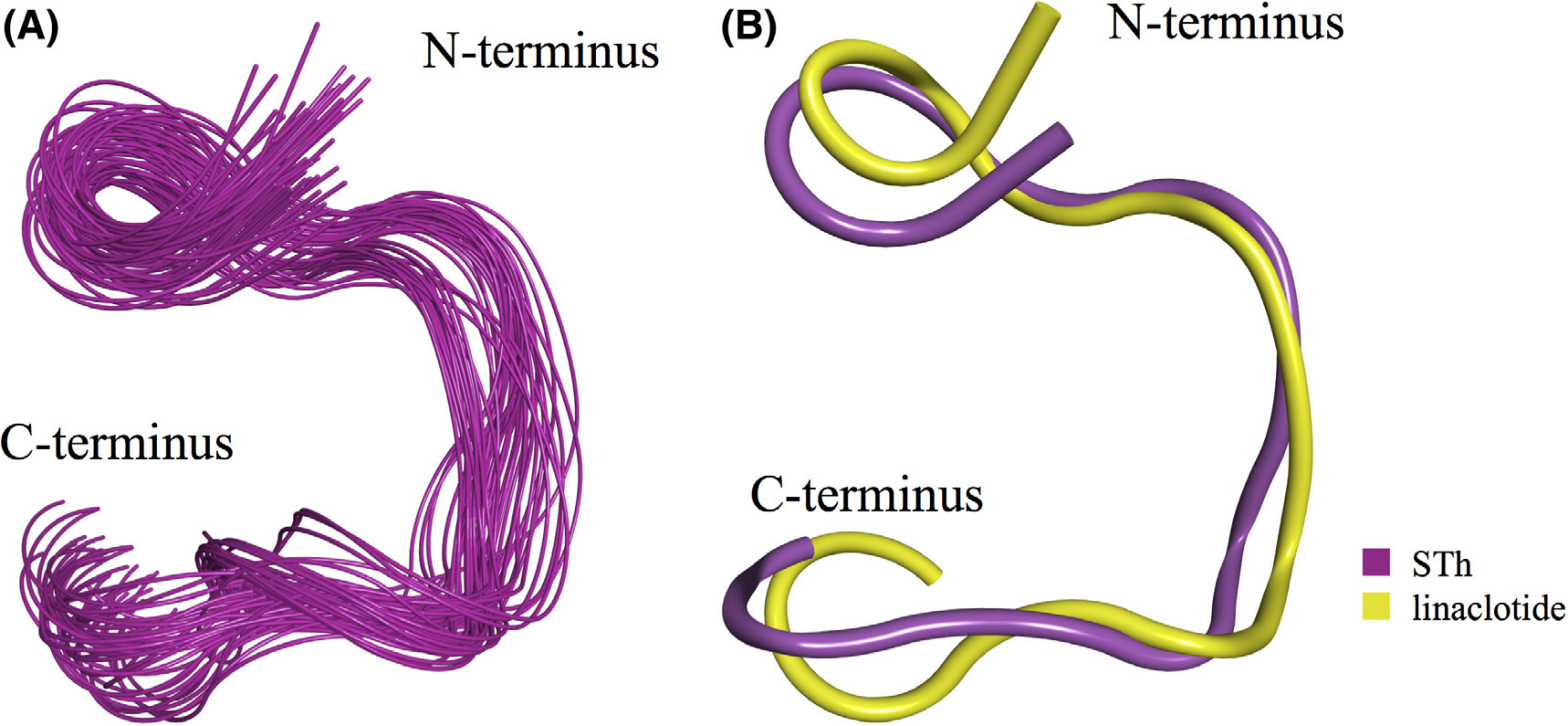
MD simulations of STh and linaclotide. (A) Overlapping snapshots of STh from MD simulations reveal that the peptide adopts a single stable structure with little flexibility. (B) Superimposition of representative structures from the STh and linaclotide. Variations between structures at the C‐terminus reflects changes in conformation induced by the additional tyrosine of linaclotide. Simulations reveal that linaclotide adopts a similar conformation as STh especially so within the region of the GC‐C interaction loop. (*). MD, Molecular dynamics.

In addition to providing information on structure, the simulations also provided information on the degree of internal rigidity and flexibility within the peptide. This can be observed in the MD simulations shown in Figure 2A, in which overlapping snapshots of STh show very little deviation. The fact that the snapshots show a high degree of overlap across all rounds of simulations indicates that STh is a fairly rigid molecule with little internal flexibility. This constrained geometry is thought to be established by the three disulfide bonds of the peptide leading to a structural rigidity and high binding affinity of STh for the GC‐C receptor (Ozaki et al. 1991).

MD simulations of linaclotide, which differs from the STh peptide used in the simulations by one amino acid substitution and an additional residue at the C‐terminus, also reveal a rigid molecule that adopts a similar conformation as STh (Fig. 2B). The rigidity of this peptide was also confirmed by a RSM fluctuation analysis of the MD trajectory (Supplemental Information, Fig. S2). Some variability can be seen within the C‐termini of the peptides, likely due to the extra amino acid in linaclotide compared to STh. Moreover, the conformation of the interaction loops (regions binding the GC‐C receptor) is homologous between the two molecules (Fig. 2B *) (Ozaki et al. 1991).

### MD simulations of plecanatide

MD simulations of plecanatide were conducted by altering the amino acid sequence of the NMR structure of uroguanylin (Marx et al. 1998). Because pH has been shown to alter the ability of uroguanylin to activate GC‐C receptors, simulations were conducted on the four ionization states of plecanatide's structure, reflecting three different pH values, by altering the protonation states of Asp2 and Glu3 residues. It should be noted that plecanatide contains an additional pH‐sensitive residue, Glu5. However, unlike Glu3, its side chain is oriented away from the interaction loop and, given its position between the two disulfide bonds, Glu5 does not have the conformational freedom to affect the orientation of the loop itself. Furthermore, this specific residue is highly conserved across the whole range of GC‐C binding peptides, and includes STh and linaclotide which are not affected by pH variations (Busby et al. 2010). For these reasons, the protonation state of Glu5 should not affect the activity of uroguanylin and plecanatide.

**Figure 3:**
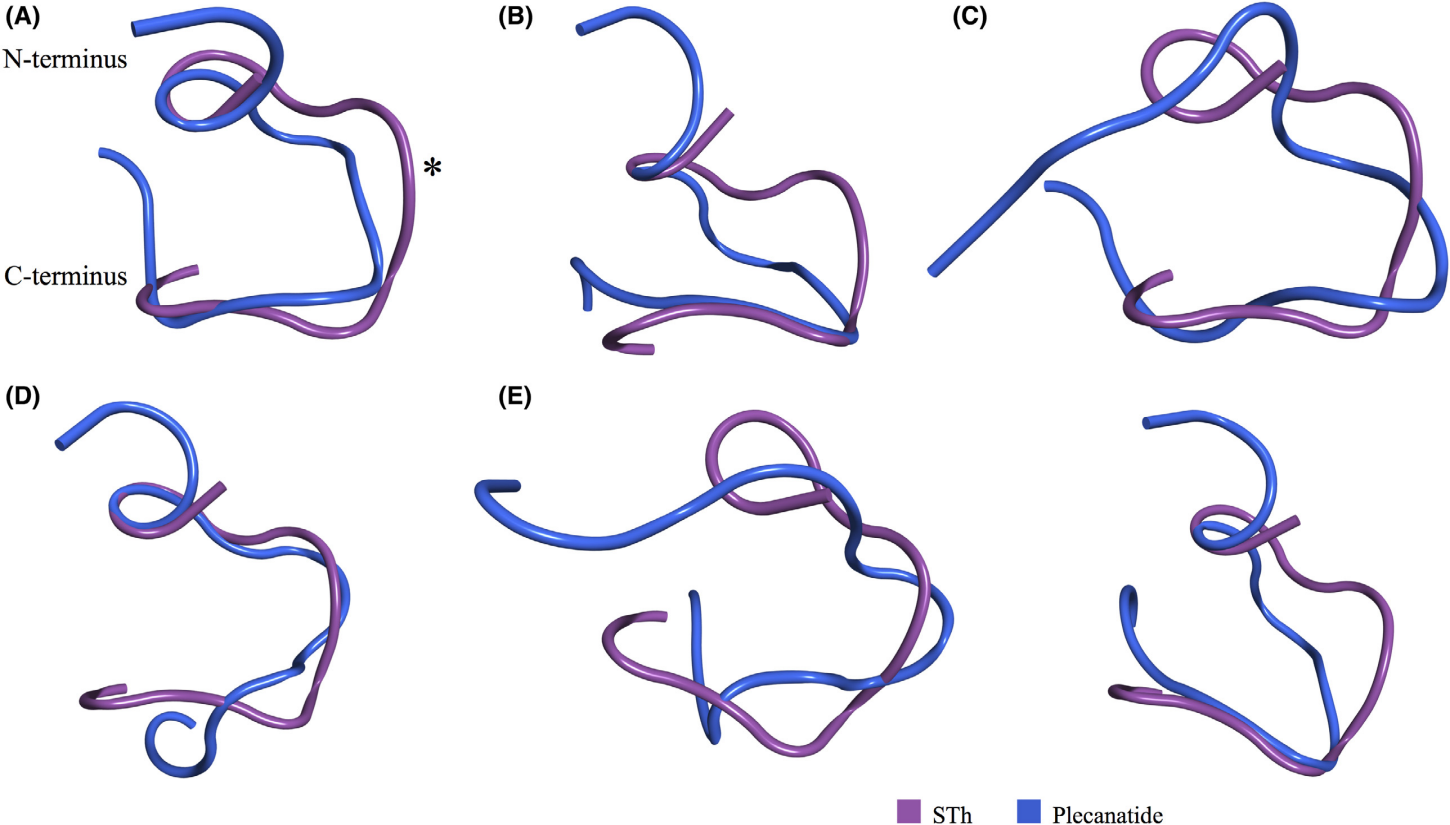
Overlapping snapshots of STh and plecanatide at pH values 5.0 (A–D), 2.0 (E) and >7.0 (F) using MD simulations. (A–C) Overlay of the three predominant Asp‐/Glu conformations of plecanatide and STh at pH 5.0 (RMSD values – A: 2.38 Å; B:2.48 Å; C:3.44 Å). (D) The predominant Asp/Glu‐ conformation of plecanatide and STh at pH 5.0 (RMSD value–1.93 Å). The overlapping conformation of the interaction loops (*) in A and D suggest these are active forms of plecanatide able to bind to and stimulate GC‐C receptors. (E) Plecanatide conformations at pH 2.0 Asp/Glu differ from those of STh with no overlap of interaction loop (RMSD value–3.45 Å). (F) Simulations of the double negative form of plecanatide, Asp‐/Glu‐, representing the protonation state at pH > 7.0, reveal a single plecanatide structure that has a minimal overlap of the interaction loop (RMSD value – 2.54 Å). MD, Molecular dynamics.

To represent plecanatide at pH 5.0, which corresponds to the pH of the duodenum and proximal jejunum, two protonation configurations were analyzed. In one, Asp2 is protonated (Asp/Glu‐), whereas in the other, Glu3 is protonated (Asp‐/Glu). These ionization states were based on the consideration that, as these residues are on the flexible N‐terminus and exposed to solvent, their pKa values would be between 3.5 and 4.5 (values dependent on the input peptide conformation as calculated on http://biophysics.cs.vt.edu/H++, version 3.2) (Anandakrishnan et al. 2012); hence, the monoprotonated states would likely be present at pH 5.0. Simulations of the two protonation states indicate that plecanatide is flexible at this pH and can adopt several conformations. Figures 3A–C show an overlay of the three predominant Asp‐/Glu conformations of plecanatide and STh, and Figure 3D shows the predominant Asp/Glu‐ conformation of plecanatide and STh. In two of these structures (Fig. 3A and D), the interaction loop (*) of plecanatide overlays well with that of STh, indicating that these two conformations of plecanatide are capable of binding to and subsequently activating GC‐C receptors.

Simulations of the double protonated form of plecanatide (Asp/Glu) were conducted to assess the structure and dynamics of the peptide at pH 2.0. The plecanatide conformations observed at this pH differ from those of STh (Fig. 3E). Based on these results, plecanatide is unlikely to adopt a conformation capable of binding to GC‐C at this highly acidic pH level, a feature which would mimic uroguanylin's inability to activate GC‐C receptors at this pH.

Simulations of the double negative form of plecanatide (Asp‐/Glu‐), which represent the protonation state observed at pH > 7.0, reveal a single predominant plecanatide structure (Fig. 3F). The portion of the peptide that interacts with the GC‐C receptor adopts a different conformation in plecanatide than in STh indicating diminished activity of the molecule at this pH value.

**Figure 4:**
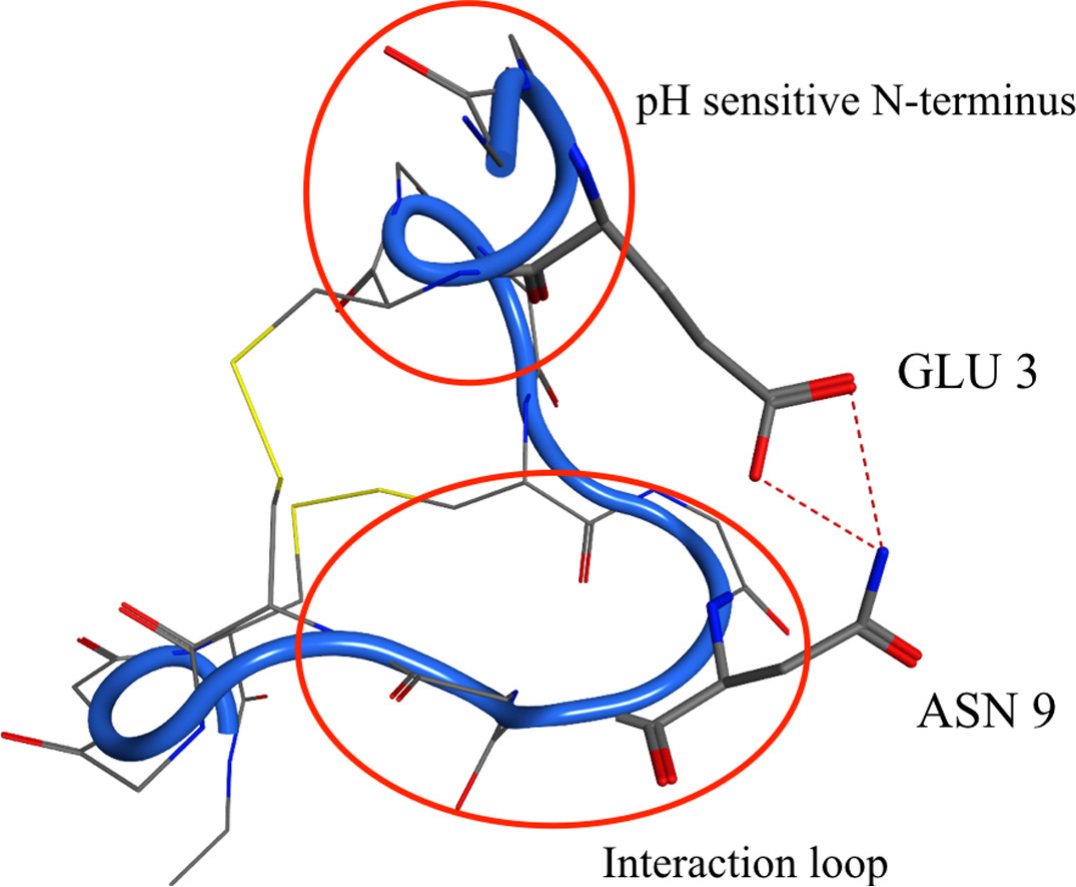
Structure of plecanatide at pH 5.0, in the Asp/Glu‐ ionization state. Interaction (*) between the negative charge of Glu3 residue on the pH‐sensitive N‐terminus and Asn9 residue in the interaction loop potentially serves to stabilize this active conformation of plecanatide.

Interestingly, in the Asp/Glu‐ ionization state of plecanatide at pH 5, an interaction occurred between the negatively charged acidic side chain of Glu3 residue in the N‐terminus and the positively charged side chain of Asn9 in the interaction loop (Fig. 4). This interaction between the Glu3 residue of the N‐terminus and the Asn9 residue of the interaction loop seems to stabilize plecanatide in its most active conformation at pH 5. This interaction was not observed at other pH values nor is it expected to occur with uroguanylin as the Asp3 amino acid in uroguanylin would not be of sufficient length to interact with the Asn9 residue in its interaction loop.

**Figure 5:**
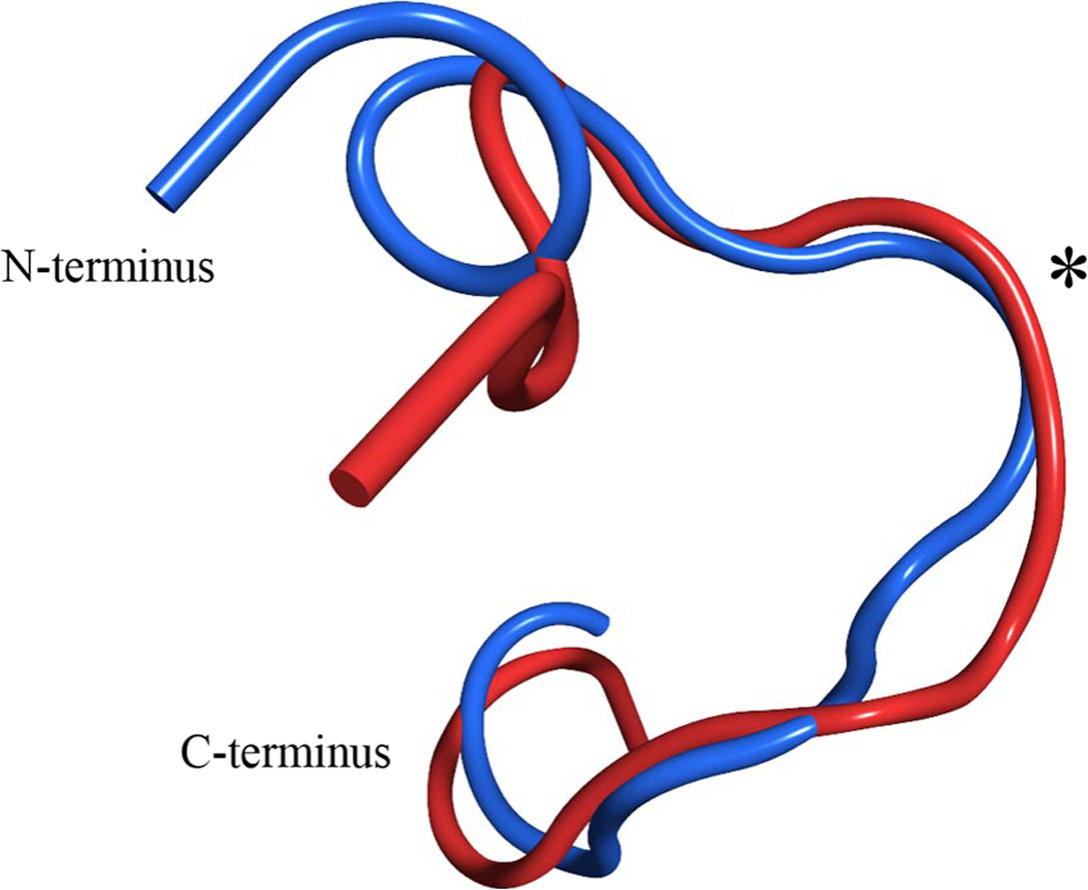
Overlay of the most active conformation of plecanatide, Asp/Glu‐, (pH 5.0), with the active “A” NMR structure of uroguanylin. The interaction loop of the two peptides overlaps generally very well (*). Some variability is observed in the N‐terminus, where the substitution Asp3/Glu3 is present.

Overall, the highly flexible behavior of plecanatide is very similar to the one observed with the parent peptide uroguanylin (Fig. 5). Indeed, the NMR structures available for the latter peptide show a high degree of conformational variability that closely mimics plecanatide. The RMS Fluctuation analysis on Plecanatide also confirms this flexibility (Fig. S2). The interaction loop of the two peptides overlaps generally very well. Some variability is observed in the N‐terminus, where the substitution of Asp3 for Glu3 is present.

In summary, if we consider all simulation sets at pH 2.0, 5.0, and 7.0, we can observe that plecanatide has the required flexibility at these pH values to switch between numerous conformations. The most active forms of plecanatide were found with the two ionization states representative of pH 5.0 as revealed by the similarity of their interaction loops with the corresponding regions in uroguanylin at pH 5 (Fig. 5) and STh (Fig. 3A and F). These results are consistent with previous studies reporting that plecanatide was designed to be more active at slightly acidic pH values, mimicking the pH‐sensitive behavior of uroguanylin (Shailubhai et al. 2013).

### Comparison of MD simulations

RMSD comparisons of the different peptides analyzed were used to quantify the similarities between the structures. The STh structure was used as a reference and the conformations of the different peptides were superimposed on the STh molecule to evaluate their similarity to the prototype of the active peptide, with lower RMSD values indicating more similarity. Table 2 shows the RMSD value for each peptide, considering all the representative clusters. With an RMSD of 1.28 Å, the structure of linaclotide is more similar to STh than to uroguanylin or to any of the plecanatide variants. The RMSD values of the different plecanatide conformations show how flexible the peptide is. Interestingly, plecanatide presents some conformations that are very close to the reference STh (RMSD <2 Å), suggesting a peak activity for plecanatide at this pH. Additionally, RMSD calculations reveal that these four variants of plecanatide are the closest structurally to the active A‐form of uroguanylin. This is consistent with the data described above, in which the negative charge of Glu3 interacts with Asn9 in the interaction loop, potentially promoting an active conformation of plecanatide. These results indicate that, in a manner similar to uroguanylin, the activity of plecanatide is not “all or nothing” but tunable based upon the pH of the environment.

**Table 2:** RMSD comparison between STh and the most representative clusters of the different peptides simulated

Compound and Test Condition	RMSD–Å
Linaclotide	1.28
Uroguanylin	1.2–2.34^a^
Plecanatide ‐ pH>7.0	2.54
Plecanatide – pH 5.0 (Asp‐/Glu)	3.44, 2.48, 2.38^b^
Plecanatide – pH 5.0 (Asp/Glu‐)	1.93
Plecanatide – pH<2.0	3.45

a Range of RMSD values between STh and the 10 NMR conformations of uroguanylin.

b Three representative clusters (>10% – see methods section) were obtained for this peptide. RMSD comparisons of the different peptides analyzed were used to quantify the similarities between the structures. Lower values indicate higher structural similarity with STh reference molecule.

## Discussion

Transmembrane GC‐C receptors play a critical role in the regulation of fluid and electrolyte homeostasis within the GI tract (Brierley 2012; Steinbrecher 2014). This balance is normally maintained by the pH‐dependent activation of these receptors by the endogenous ligands, uroguanylin, and guanylin (Forte 2004). Studies have shown that these pH environments are maintained at differential values across the length of the gastrointestinal tract (Daniel et al. 1985; Whitaker et al. 1997). In the slightly acidic mucosal environments of the duodenum and proximal jejunum (pH 5.0‐6.0), uroguanylin is 10 times more potent than in the slightly basic (pH 7.0‐8.0) environments of the ileum and colon (Hamra et al. 1997). In contrast, guanylin is significantly more potent at binding GC‐C receptors in the ileum and colon (pH 7.0‐8.0) than in the duodenum and proximal jejunum (pH 5.0‐6.0), where it is essentially inactive (Hamra et al. 1997). Structural shifts induced by pH environments alter the potency of these ligands, allowing them to cooperatively regulate GC‐C receptor activation (Hamra et al. 1997). Any perturbation in the pH‐dependent control of GC‐C activation would disrupt the delicate balance of electrolytes and fluids within the intestines. This disruption could be associated with disorders such as obstruction, secretory diarrhea and inflammatory bowel disease (Field 2003; Fiskerstrand et al. 2012).

Based on their physiology, GC‐C receptors are a promising therapeutic target in the treatment of numerous gastrointestinal disorders such as CIC and IBS‐C. Both are common complaints among all age groups and are associated with a substantial burden on healthcare resources in the US (Drossman et al. 2009). While the exact pathologic mechanism of these diseases remains an area of intense study, the symptoms (i.e. diminished stool frequency, hard/lumpy stools, straining abdominal symptoms) indicate an imbalance in fluid and electrolyte homeostasis in the GI tract (Steinbrecher 2014).

As an analog of the pathological GC‐C agonist STh, linaclotide maintains many structural features of STh, including the presence of three disulfide bonds and an insensitivity to pH. MD simulations in this study show that the addition of a third intramolecular bond makes both STh and linaclotide insensitive to MD perturbations (Ozaki et al. 1991). The structural similarity of these two molecules is reflected by the low RMSD values of 1.28 Å for linaclotide. The amino acid substitutions that differentiate linaclotide from STh further enhance the pharmacokinetic stability and proteolytic resistance of linaclotide, allowing it to remain active across a longer portion of the small intestine (Bharucha and Waldman 2010; Harris and Crowell 2007). The absence of pH‐sensing amino acid residues would additionally give these molecules maximum biological activity across the range of pH environments in the GI tract. This lack of focused areas of activity may induce excessive fluid secretion and explain the increased incidence of diarrhea associated with linaclotide (Busby and Ortiz 2014; Carpick and Gariepy 1993; Lembo et al. 2011).

Plecanatide, a GC‐C agonist under investigation for IBS‐C and CIC, differs from linaclotide in its amino acid sequence and number of disulfide bonds. Plecanatide maintains many of the structural and functional characteristics of endogenous ligand uroguanylin, including the two disulfide bonds and the two N‐terminal pH‐sensing acidic residues, which result in a molecule that can exhibit differential levels of activity based upon the pH of the environment. As a result, plecanatide has a more targeted zone of activation, stimulating GC‐C receptors under acidic conditions in a way that is similar to uroguanylin (Shailubhai et al. 2015).

MD simulations of plecanatide in this study revealed plecanatide to be a flexible structure that is unlike STh or linaclotide and one that is able to adopt numerous conformations in response to a range of simulated pH environments. Optimally active conformations were observed in pH 5.0 simulations which approximates the pH values of the duodenum and proximal jejunum. This pattern of optimal activity is similar to that of uroguanylin, indicating that plecanatide and uroguanylin are similar in both structure and function (Shailubhai et al. 2013). In contrast, at simulated pH values of 2.0 and 7.0, plecanatide adopted conformations that would make it less likely to bind GC‐C receptors. Across the range of assessed pH values, MD simulations and RMSD analysis reveal that the structure of plecanatide and the NMR structure of uroguanylin are similar to each other (Fig. 5), and quite dissimilar to the STh structure (1.93–3.45 Å and 1.20–2.34 Å vs. STh, respectively).

As mentioned previously, the sole difference between the structures of uroguanylin and plecanatide is the replacement of an aspartic acid residue (Asp3) with a glutamic acid residue (Glu3) in plecanatide (Fig. 1). Previous studies have shown that while plecanatide has a similar pH‐dependent affinity for the GC‐C receptor, it binds with superior potency than uroguanylin (Shailubhai et al. 2000, 2015). Computational models in this study revealed that at pH 5.0, the charged end of the Glu3 residue interacts with the Asn9 residue, potentially serving to stabilize the structure when it forms its most active conformation. Interestingly, the corresponding interaction between Asp3 and Asn9 is not present in uroguanylin, probably because the side chain of Asp3 is not long enough to reach Asn9. Stabilizing the molecule in this way allows plecanatide to remain in its active conformer for a longer period of time at this pH and explain the superior binding profile of plecanatide versus uroguanylin witnessed in earlier studies.

Recognizing the acidic‐to‐basic pH gradient nature of the GI tract, the simulations in this study suggest that, similar to uroguanylin, plecanatide would be optimally active in the more acidic environment that is present in the proximal small intestine of the GI tract. The ability of plecanatide to activate GC‐C receptors in a targeted manner similar to the endogenous ligand uroguanylin may help normalize bowel movements and explain the low levels of adverse events seen with the drug in early clinical trials (Shailubhai et al. 2013). Understanding the structural mechanisms by which plecanatide and linaclotide activate GC‐C receptors and respond to pH could shed light on clinical differences between the two therapeutic options.

## Authorship Contributions

Brancale and Shailubhai participated in research design. Ferla, Ricci, and Bassetto conducted the experiments. Brancale and Ferla performed data analysis. Brancale, Shailubhai, and Jacob wrote or contributed to the writing of the manuscript.

## Disclosures

K Shailubhai and G S Jacob are employees and stockholders of Synergy Pharmaceuticals.

## Supporting information


**Figure S1.** The graphs represent the RMSD variation during the simulation for each peptide. The RMSD is calculated taking as reference the structure of the most representative cluster for each peptide. In the case of Plecanatide‐pH>5.0 (Asn‐/Glu), three major clusters were present. All the frames included in the interval between 0 and 0.1 Å are part of the representative clusters for each peptide.
**Figure S2.** Residues RMS fluctuation. It is clear how the fluctuation is minimal for STh and Linaclotide due to the higher rigidity of the two peptide. On the contrary, a higher flexibility is obtained for the four Plecanatide peptides, according to the different protonation states.
