# Supplementary material for: Therapeutically targeting guanylate cyclase‐C: computational modeling of plecanatide, a uroguanylin analog
Source: Pharmacol Res Perspect. 2017 Mar 12;5(2):e00295. doi: 10.1002/prp2.295 (PMC5368960; doi:10.1002/prp2.295)
Supplement: Supplementary file 1 — Figure S1. The graphs represent the RMSD variation during the simulation for each peptide. The RMSD is calculated taking as reference the structure of the most representative cluster for each peptide. In the case of Plecanatide‐pH>5.0 (Asn‐/Glu), three major clusters were present. All the frames included in the interval between 0 and 0.1 Å are part of the representative clusters for each peptide. Figure S2. Residues RMS fluctuation. It is clear how the fluctuation is minimal for STh and Linaclotide due to the higher rigidity of the two peptide. On the contrary, a higher flexibility is obtained for the four Plecanatide peptides, according to the different protonation states. [file PRP2-5-e00295-s001.docx]

Therapeutically Targeting Guanylate Cyclase-C: Computational Modeling of Plecanatide, a Uroguanylin Analog

Brancale A^1^, Shailubhai K^2^, Ferla S^1^, Ricci A^1^, Bassetto M^1^, Jacob G S^2^

^1^ School of Pharmacy and Pharmaceutical Sciences, Cardiff University

^2^ Synergy Pharmaceuticals, New York, NY

Primary Laboratory:

Cardiff School of Pharmacy and Pharmaceutical Sciences

Cardiff University, Redwood Building
King Edward VII Avenue, Cardiff, CF10 3NB

Supplemental Information


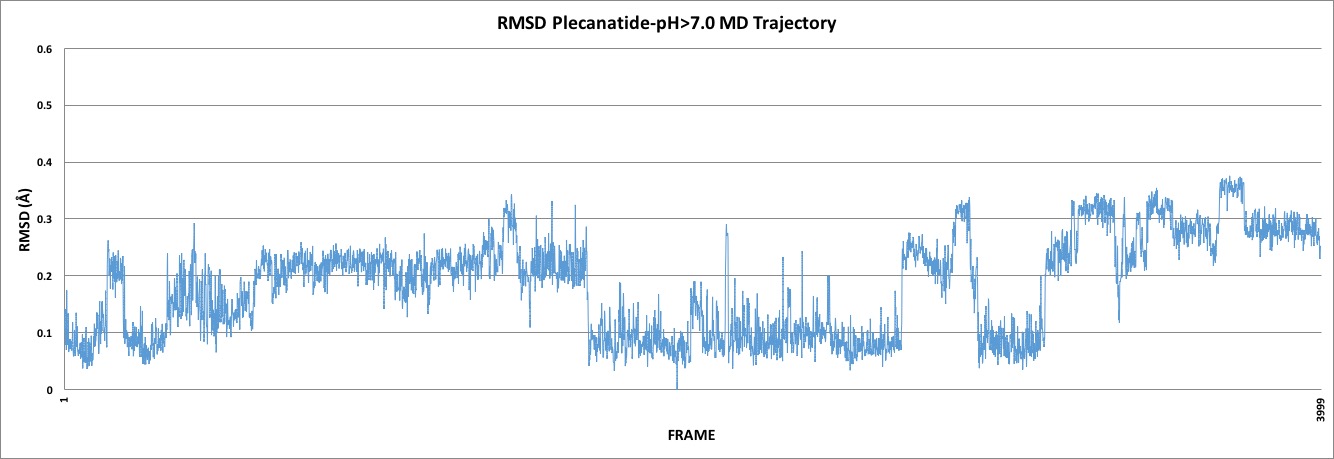


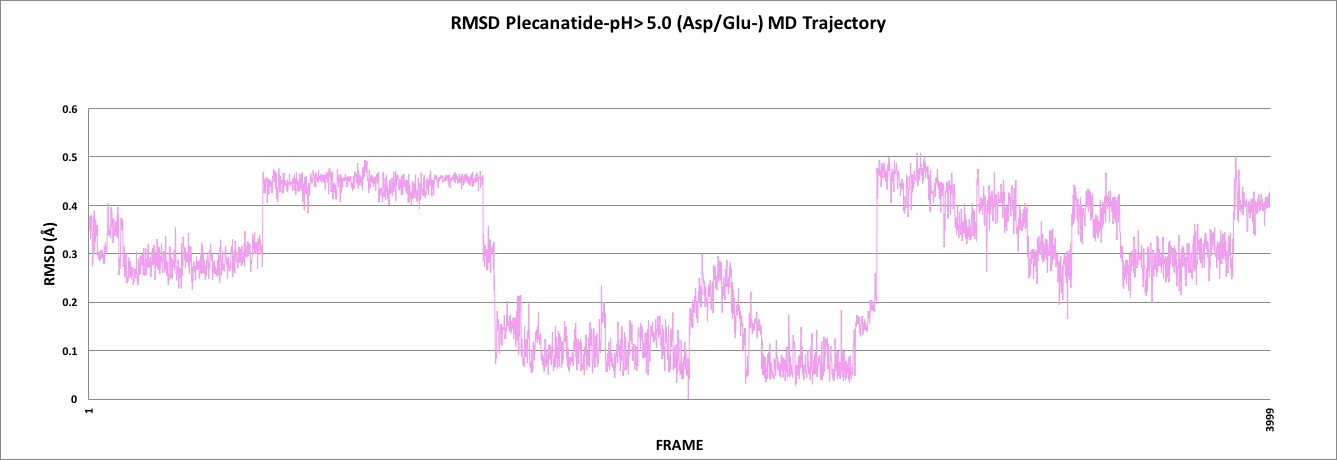


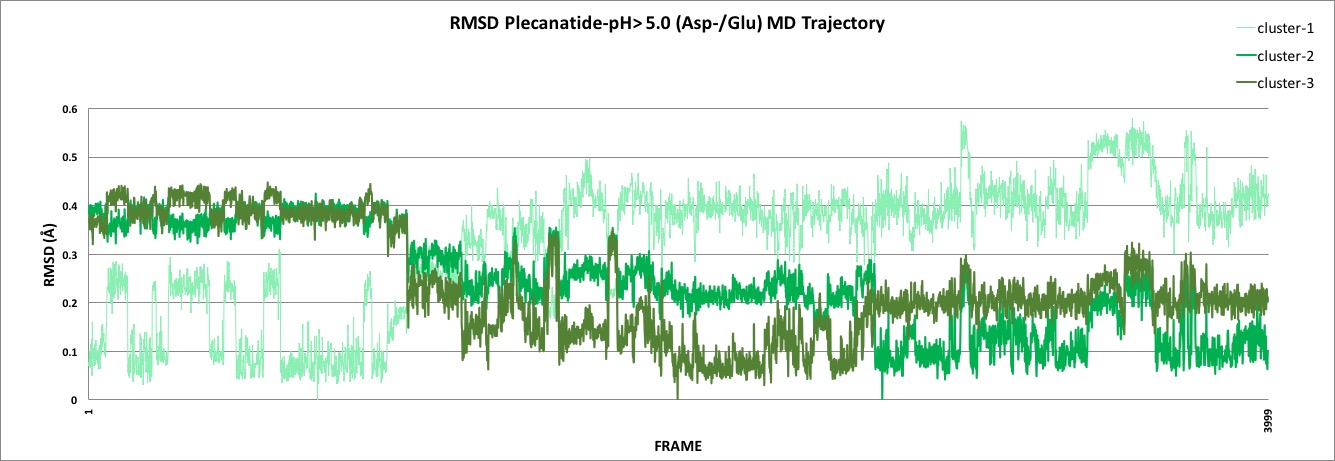


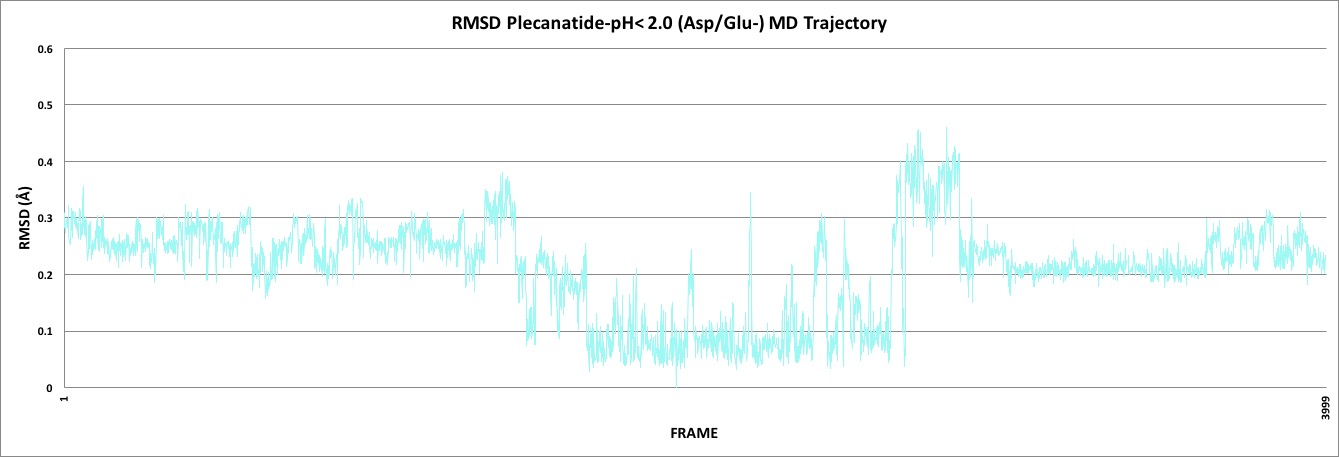


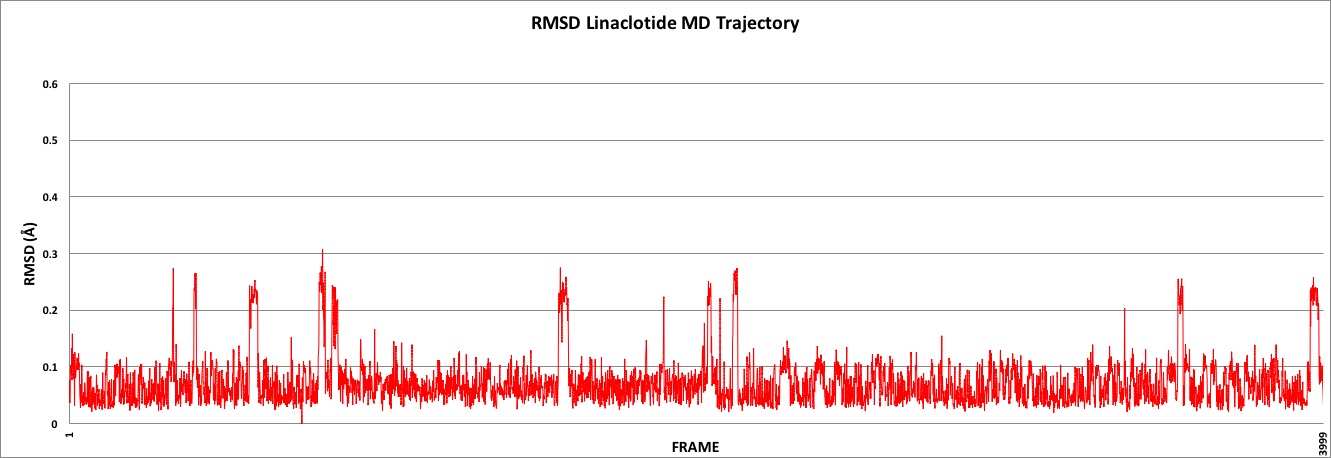


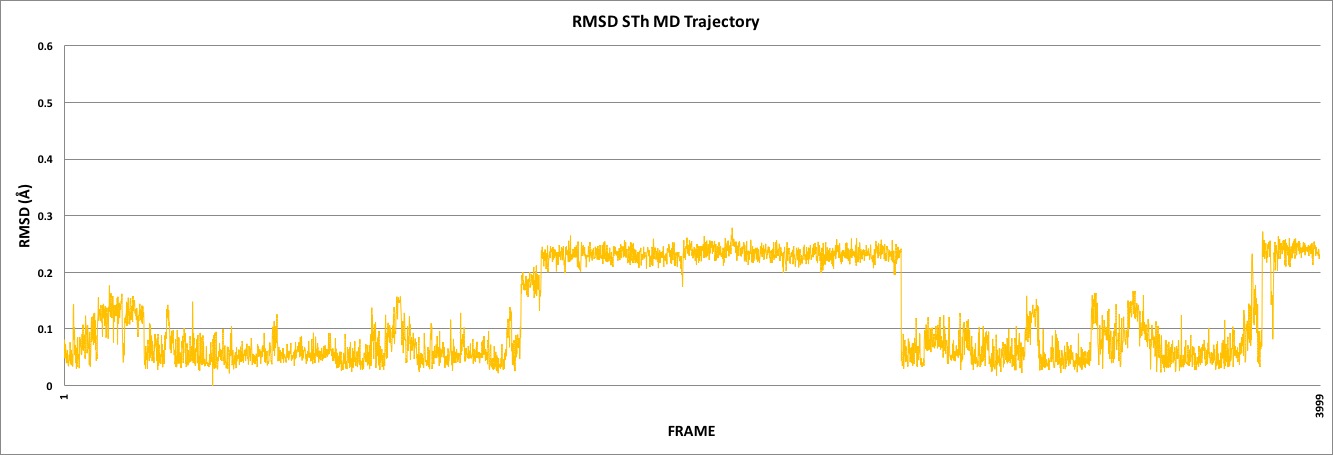


**Supplemental Figure 1.** The graphs represent the RMSD variation during the simulation for each peptide. The RMSD is calculated taking as reference the structure of the most representative cluster for each peptide. In the case of Plecanatide-pH>5.0 (Asn-/Glu), 3 major clusters were present. All the frames included in the interval between 0 and 0.1 Å are part of the representative clusters for each peptide.


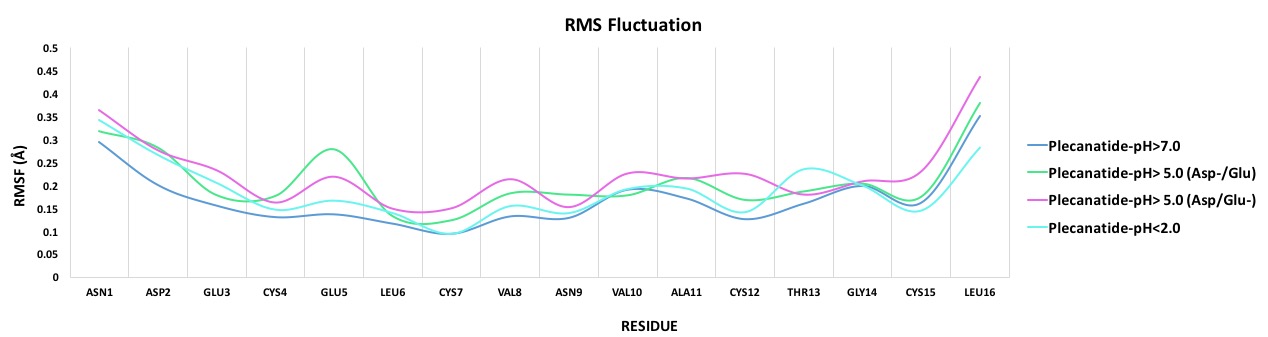


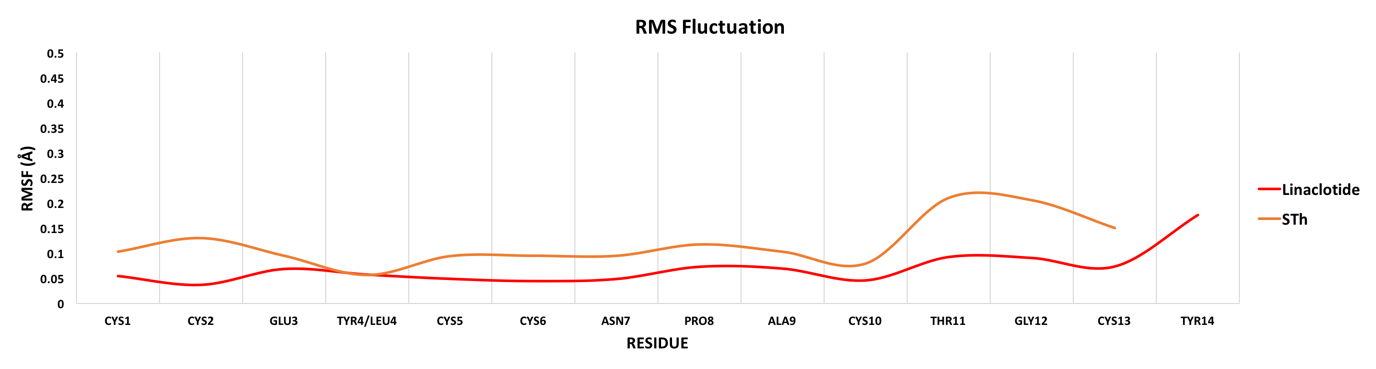


**Supplemental Figure 2.** Residues RMS fluctuation. It is clear how the fluctuation is minimal for STh and Linaclotide due to the higher rigidity of the two peptide. On the contrary, a higher flexibility is obtained for the four Plecanatide peptides, according to the different protonation states.
